# Mechanisms of Intestinal Serotonin Transporter (SERT) Upregulation by TGF-β1 Induced Non-Smad Pathways

**DOI:** 10.1371/journal.pone.0120447

**Published:** 2015-05-08

**Authors:** Saad Nazir, Anoop Kumar, Ishita Chatterjee, Arivarasu N. Anbazhagan, Tarunmeet Gujral, Shubha Priyamvada, Seema Saksena, Waddah A. Alrefai, Pradeep K. Dudeja, Ravinder K. Gill

**Affiliations:** 1 Division of Gastroenterology & Hepatology, Dept. of Medicine, University of Illinois at Chicago, Chicago, Illinois, United States of America; 2 Jesse Brown VA Medical Center, Chicago, Illinois, United States of America; UCSF / VA Medical Center, UNITED STATES

## Abstract

TGF-β1 is an important multifunctional cytokine with numerous protective effects on intestinal mucosa. The influence of TGF-β1 on serotonin transporter (SERT) activity, the critical mechanism regulating the extracellular availability of serotonin (5-HT), is not known. Current studies were designed to examine acute effects of TGF-β1 on SERT. Model human intestinal Caco-2 cells grown as monolayer’s or as cysts in 3D culture and ex vivo mouse model were utilized. Treatment of Caco-2 cells with TGF-β1 (10 ng/ml, 60 min) stimulated SERT activity (~2 fold, P<0.005). This stimulation of SERT function was dependent upon activation of TGF-β1 receptor (TGFRI) as SB-431542, a specific TGF-βRI inhibitor blocked the SERT stimulation. SERT activation in response to TGF-β1 was attenuated by inhibition of PI3K and occurred via enhanced recruitment of SERT-GFP to apical surface in a PI3K dependent manner. The exocytosis inhibitor brefeldin A (2.5 μM) attenuated the TGF-β1-mediated increase in SERT function. TGF-β1 increased the association of SERT with the soluble N-ethylmaleimide-sensitive factor attachment protein receptor (SNARE) syntaxin 3 (STX3) and promoted exocytosis of SERT. Caco-2 cells grown as cysts in 3D culture recapitulated the effects of TGF-β1 showing increased luminal staining of SERT. Ussing chamber studies revealed increase in ^3^H-5-HT uptake in mouse ileum treated ex vivo with TGF-β1 (10 ng/ml, 1h). These data demonstrate a novel mechanism rapidly regulating intestinal SERT via PI3K and STX3. Since decreased SERT is implicated in various gastro-intestinal disorders e.g IBD, IBS and diarrhea, understanding mechanisms stimulating SERT function by TGF-β1 offers a novel therapeutic strategy to treat GI disorders.

## Introduction

Accumulating evidence indicates that increased availability of 5-HT in the gut mucosa is involved in pathogenesis of intestinal inflammation or diarrheal disorders. Regulation of extracellular availability of 5-HT in the GI tract is dependent upon three distinct processes involving i) synthesis of 5-HT by the action of tryptophan hydroxylase-1 (TPH-1) in enterochromaffin cells; ii) action of released 5-HT on various receptor subtypes present in various cell types such as dendritic cells, lymphocytes, macrophages, endothelial cells, and intestinal epithelial cells (IECs) and iii) deactivation of 5-HT caused by its uptake via SERT expressed apically in IECs and its subsequent degradation by intracellular enzymes such as monoamine oxidases. Reducing the availability of 5-HT by deletion of TPH-1 in mice has been shown to reduce the severity of DSS induced colitis [1]. Similarly, blockade of 5-HT3 receptor subtypes exerted beneficial effects in colitis in rats [2]. However, mechanisms underlying modulation of SERT, a critical mediator regulating 5-HT availability, have not been fully investigated and may represent a novel approach to restore 5-HT homeostasis in the treatment of both infectious and inflammatory disorders of the intestine.

In this context, growth factors have lately emerged as promising interventions for treatment of intestinal inflammation and repair in animal models and human subjects. For example, short-term treatment with epidermal growth factor (EGF) enemas has proven beneficial in ulcerative colitis patients [3]. TGF-β1, in particular, is an important multifunctional cytokine and a potent negative regulator of intestinal inflammation [4, 5]. Under physiological conditions, TGF-β1 aids in epithelial defense mechanisms, regulates proliferation, extracellular matrix turnover, differentiation and function of immune and non-immune cells [5]. Evidence from a variety of *in vivo* animal models has demonstrated that eliminating TGF-β1 or its downstream signaling cascade leads to inflammatory disease [6–8]. Indeed, a distinguishing feature of TGF-β1 knock out mice is the uncontrolled multifocal inflammation with extensive inflammatory infiltrates including the intestine [6]. Similarly, loss of TGF-β1 signaling specifically in the intestine (utilizing transgenic mice expressing dominant negative form of TGF-β type II receptor) contributed to the development of colitis [9]. In parallel, exogenous administration of TGF-β1 has proven effective in ameliorating inflammatory responses [4, 10]. However, therapy using growth factors over-a longer-periods of time might pose a concern with regards to development of malignant transformation with EGF or fibrosis with TGF-β1 [3, 11]. Therefore, an understanding of cellular and molecular mechanisms underlying effects of growth factor is, thus, critical to yield valuable insights into development of novel and better targets for treatment modalities. Our previous studies demonstrated that EGF upregulates SERT via transcriptional mechanisms [12]. Whether TGF-β1 modulates SERT is not known and warrants extensive investigation.

TGF-β1 initiates a signaling cascade via activation of a heterodimeric complex of type I and type II transmembrane receptors (TGFRI and TGFRII). Ligand binding to the TGFPII causes activation of TGFRI, which in turn phopshorylates a set of proteins called Smads that translocate to the nucleus to execute transcriptional activation or repression of responsive target genes [13]. There are alternate non-Smad signaling pathways induced by TGF-β1 including, PI3K pathway, extracellular signal-regulated kinase (Erk1/2), MAPK (p38) and Src dependent pathways [14]. Since mechanisms that respond to exogenous stimuli and rapidly modulate SERT activity are not fully identified, we first focused our attention to non-Smad pathways in short-term modulation of SERT by TGF-β1. Our studies, for the first time, demonstrate that TGF-β1 acutely stimulates SERT function via TGF-β1 receptor activated non-Smad pathways in IECs. These studies highlight a critical role of membrane trafficking events dependent upon PI3K in rapidly increasing the activity and recruitment of SERT to apical surface by enhancing its exocytosis and association with SNARE protein STX3.

## Materials and Methods

### Materials

Caco-2 cells were obtained from ATCC. [^3^H] 5-HT-HCl was obtained from Perkin Elmer (Waltham, MA). Recombinant TGF-β1 was obtained from Sigma Aldrich (St Louis, MO). SERT antibody was procured from Abcam (Cambridge, MA). Syntaxin 1A and 3 antibodies were from Cell Signaling.

### Models

#### 2D-Cell culture

Caco-2 cells were grown in T-75 cm^2^ plastic flasks at 37°C in a 5% CO_2_/95% air environment. The culture medium consisted of high-glucose MEM, 20% FBS, 20 mM HEPES, 100 IU/ml penicillin, and 100 μg/ml streptomycin. Cells used for these studies were between passages 25 and 45. Cell monolayers grown on collagen coated Transwell inserts were plated at a density of 1X10^4^ cells/well for 10–12 days and used at day 12 post-plating. For treatment, cell monolayers were serum-starved overnight and treated from basolateral side with TGF-β1 (10ng/ml) in serum-free medium supplemented with 0.2% BSA.

#### 3D-Cell culture

To culture Caco2 cells in 3-dimensional system, 8-well chamber slides were pre-coated with 30 l of matrigel (BD biosciences). Caco2 cells were then plated at a density of 4000 cells/in each well of 8-well and allowed to solidify for 10 min at 37°C, then overlayed with 400 μl of Eagle's medium (EMEM) supplemented with 20% FBS and penicillin—gentamycin (100 IU/ml and 100 g/ml, respectively) mixed with Hepes. Cells were subsequently maintained for 12 days at 37°C in 5% CO_2_ for differentiation and formation of cysts. For TGF-β1 treatment, 3D Caco2 cysts were kept in serum free condition for 16 h later treated with 10 ng/ml TGF-β1 for 1h then processed for RNA, protein extraction and immunostaining.

#### *Ex-vivo* model

All experiments involving mice were approved by Animal Care Committee of the University of Illinois at Chicago and Jesse Brown VA Medical Center. C57BL/6 mice (6 to 7 weeks) were procured from Jackson laboratories. After one week of acclimation, animals were euthanized and small intestine was isolated for further studies. For performing ex vivo experiments, each experimental group included minimum of 3–4 animals.

### Transient transfections and fixed cell imaging

SERT cDNA fused to GFP in mammalian expression vector cloned by us was utilized [15]. Cells were transfected with SERT-GFP vector utilizing lipofectamine. Cells, 48h post-transfection were treated with TGF-β1 (10 ng/ml) for 1h from basolateral side. During the last 10 min of incubation with TGF-β1, cells were labeled with 1 μg/ml fluorescent wheat germ agglutinin (WGA) in standard buffer (PBS), washed and fixed with 2% paraformaldehyde. Microscopy was performed utilizing Carl Zeiss LSM 510 laser scanning confocal microscope equipped with a 63 X water-immersion objective. Green and red fluorescence emissions were detected through LP485 and 585 filters, respectively. The two different fluorochromes were scanned sequentially by using multi-tracking function to avoid any bleed-through among these fluorescent dyes. A series of sections (0.5 μm) were taken at z direction and orthogonal sections were made in a z stack.

### Immunostaining in 3D culture system

For immunostaining of 3-dimensional Caco-2 culture, cysts were fixed in 2% PFA for 30 min, then treated with PBS-glycine and permeabilized with 0.5% Triton-X-100 for 15 min at room temperature (RT). After blocking, cysts were incubated in SERT antibody (1:100) for overnight in 4°C, then washed and incubated with fluorescence-conjugated secondary antibodies (1:200) (life technologies) and Phalloidin (1:200) for 45 min at RT. They were then mounted in ProLong gold antifade containing DAPI. Confocal microscopy was performed using Carl Zeiss 710 microscope.

### [^3^H] serotonin uptake in Caco-2 monolayers

Serotonin uptake experiments were performed in Caco-2 cells grown on 12-well Trans-well inserts essentially as described by us previously [15]. Untreated Caco-2 cells or cells treated with TGF-β1 were pre-incubated in medium containing 100 mM NaCl or choline chloride; 100 mM mannitol; 10 mM Tris-HEPES, pH 7.4; and 0.1 mM MgSO_4_. Serotonin uptake was initiated by the addition of 0.3 ml of medium containing 25–50 nM [^3^H] serotonin. Incubation was stopped by adding ice-cold stop solution containing 280 mM mannitol and 20 mM Tris-HEPES, pH 7.5 after 5 min. Wells were then washed with ice-cold PBS to remove extracellular [^3^H] serotonin. Cells were then lysed with 0.5 NaOH and the radioactivity was measured in a Packard TR1600 liquid scintillation counter (Packard Instruments, Downers Grove, IL). An aliquot of lysate was used for measuring protein concentration by the method of Bradford (Bio Rad Laboratories, Hercules, CA). The 5-HT uptake was expressed as pmol/mg protein/5 min.

### Cell-surface biotinylation studies

Biotinylation studies were performed to assess surface expression of SERT in Caco-2 cells endogenously expressing SERT. Cell-surface biotinylation was performed using sulfo-NH-SS-biotin (0.5 mg/ml; Pierce Biotechnology) in borate buffer (in mM: 154 NaCl, 7.2 KCl, 1.8 CaCl_2_, 10 H_3_BO_3_, pH 9.0), as previously described [15, 16]. Proteins were labeled with biotin for 60 minutes at 4°C to prevent endocytosis and internalization of antigens. After pull down of biotinylated antigens with streptavidin agarose, biotinylated proteins were released by incubation in 100 μM dithiothreitol, reconstituted in Laemmli buffer, subjected to SDS-PAGE, and then probed with anti-GFP (1:100) or anti-SERT (1:500. overnight at 4°C) purified antibody. The amount of surface SERT was compared to total cell antigen as determined by immunoblotting of solubilized cell extract and with the amount of SERT not removed by the avidin precipitation method (intracellular pool).

### Exocytosis assay

To measure exocytic insertion, cells were incubated with sulfo-NHS-acetate to saturate NHS-reactive sites on the cell surface as described previously [17, 18]. Cells were warmed *to 37°C* for 1h in the presence or absence of TGF-β1 to permit SERT recycling, surface labeled with sulfo-NHS-SS-biotin, lysed and biotinylated fraction to detect newly inserted surface SERT.

### Cell lysates, immunoprecipitation, and western blotting

Control or TGF-β1 treated Cells were washed with ice-cold PBS three times and lysed in 20 mM Tris·HCl, pH 7.5, 150 mM NaCl, 1% Triton X-100, 1 mM EDTA, 1 mM EGTA, and 1× complete protease inhibitor cocktail. The cells were homogenized by passing 10 times through 26-gauge needle. The lysate was centrifuged at 5000 *g* for 5 min at 4°C. To detect association of Syntaxin 3 with SERT, SERT was immunoprecipitated by incubating cell lysates (500 μg) with monoclonal anti-SERT overnight at 4°C, with mixing. Protein A/G plus agarose beads were added [40 μl of a 50% (wt/vol) solution] and mixed for an additional 4 h at 4°C. Immunoprecipitates were washed three times in lysis buffer, solubilized in SDS gel loading buffer, and boiled for 5 min. The samples obtained above were subjected to 10% SDS-PAGE and transferred to nitrocellulose membranes. Syntaxin 3 or SERT expression was detected utilizing specific antibodies.

### [^3^H] serotonin uptake utilizing Ussing Chambers

Ex vivo studies performed in C57BL/6J mice were approved by the Animal Care Committee of University of Illinois at Chicago and Jesse Brown Veterans Affairs Medical Center. ^3^[H] 5-HT uptake in the mouse ileum exposed to ex vivo treatment of TGF-β1 was performed as described with modifications [19]. The intestine was stripped off the muscle layer, mounted in the Ussing chamber to expose tissue on both sides to Kreb’s solution supplemented with L-ascorbic acid (100 μM) and the monoamine oxidase inhibitor pargyline (100 μM) to prevent intracellular 5-HT degradation. Briefly, tissues were treated with TGF-β1 for 1h from basolateral side and then incubated with ^3^[H]-5-HT for 30 min. Another set of ileal tissue was pre-treated with fluoxetine for 30 min before TGF-β1 treatment. ^3^[H]-5-HT in the intracellular compartments was assayed by liquid scintillation spectrometry and normalized to protein. Radioactivity was measured in a Packard TR1600 liquid scintillation counter (Packard Instruments, Downers Grove, IL).

### Statistics

Results were expressed as % of control (Mean ± SEM). Student’s t test or One-way ANOVA Tukey was used for statistical analysis. *P*<0.05 was considered statistically significant.

## Results

### Acute treatment of TGF-β1 increases SERT function via TGF-β1R

**Fig 1:**
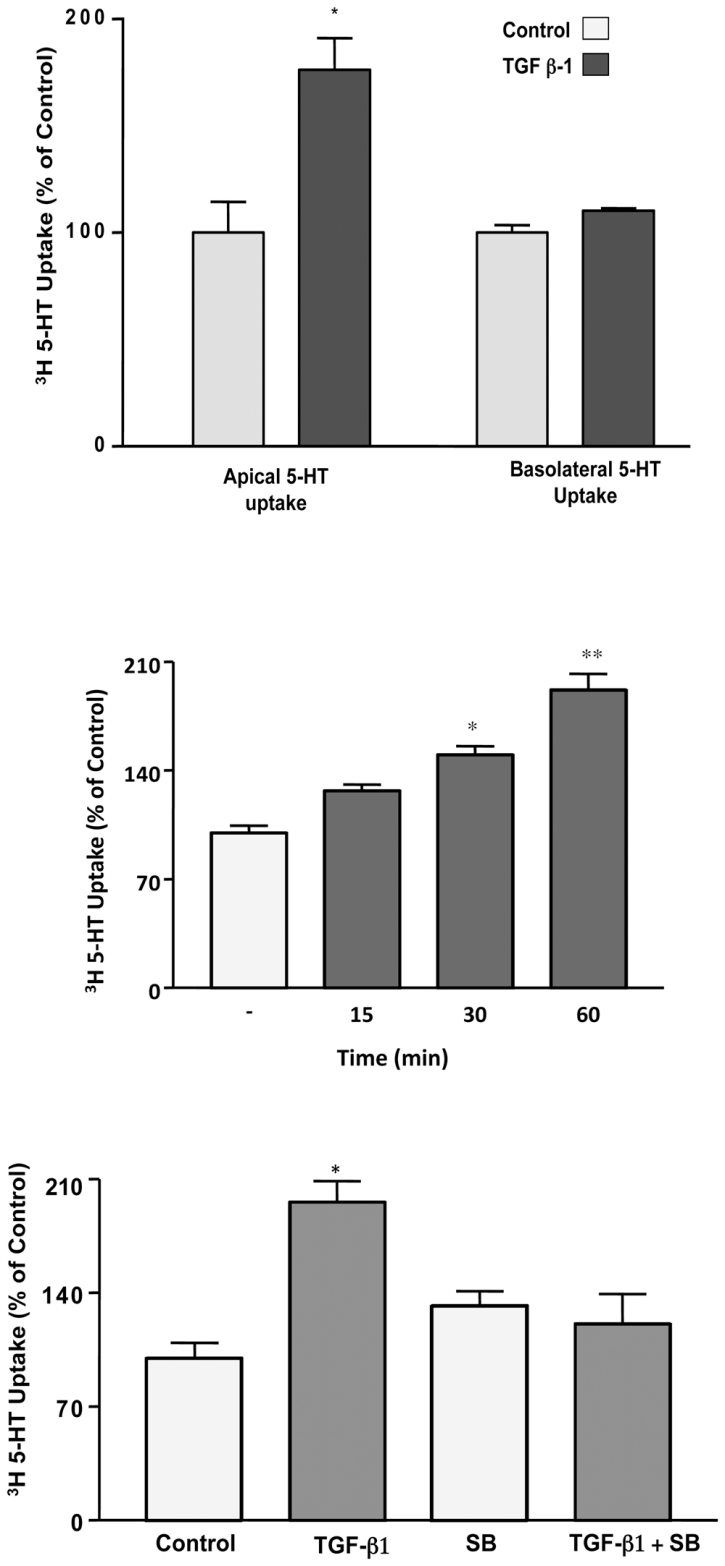
A. Short-term effects of TGF-β1 on apical and basolateral Na^+^Cl^-^-dependent ^3^[H]-5-HT uptake. Caco-2 cells were treated with TGF-β1 (10 ng/ml, 1 h) from basolateral side and SERT function was measured as ^3^[H]-5-HTuptake from either the apical side or basolateral side. [Control Values in pmol/mg protein/5 min: A) Apical uptake: 0.6 ± 0.09, Basolateral 5-HT uptake: 0.9 ± 0.03]. *P<0.05 vs control. B. Time course of TGF-β1 effects on ^3^[H]-5-HT uptake. SERT function was measured as ^3^[H]-5-HTuptake from the apical side. [Control Values in pmol/mg protein/ 5 min: 0.47 ± 0.021]. *P<0.05 vs control; ** P<0.001. C. TGF-β1 increases ^3^[H]-5-HT uptake via TGF-βR1. Cells were pre-treated from the basolateral side with the cell permeable TGFβR1 inhibitor for 1h followed by co-incubation with TGF-β1 (10ng/ml). [Control Values in pmol/mg protein/ 5 min: 0.43 ± 0.04]. N = 3–6. *P<0.05 vs control.

Caco-2 cells have been shown to exhibit functional 5-HT uptake at both apical and basolateral membranes [15, 20]. However, we have previously shown that SERT expression is predominantly restricted to the apical membranes, while the identity of the basolateral 5-HT transporter remains unknown [21]. We initiated studies to examine the short-term effects of TGF-β1 (10 ng/ml) treatment on NaCl-sensitive ^3^[5-HT] uptake in serum-starved Caco-2 cells. TGF-β1 treatment from the basolateral side stimulated apical 5-HT uptake (representing SERT activity) while the basolateral 5-HT transport was unaffected (Fig 1A). These data indicate specificity of TGF-β1 effects on apical SERT.

Depending upon the cell type, activation of signaling pathways by TGF-β1 can occur rapidly (within 5–10 min) or may require delayed response (hours) after ligand stimulation [14]. Thus, a time course of TGF-β1 (10 ng/ml) effects on apical SERT activity was first established. A significantly higher effect at 1h (~2 fold compared to control) was observed (Fig 1B).

Subsequent studies, thus, utilized 1h time point for TGF-β1 treatment. To elucidate the mechanisms of SERT stimulation by TGF-β1, the role of TGF-β1 receptors and its induced signaling pathways was investigated. TGF-β1 elicits a signaling cascade via activation of a heterodimeric complex of type I and type II Ser/Thr transmembrane receptors (TGFRI and TGFRII), localized to the basolateral side of IECs [22]. Ligand binding to the TGFPII causes recruitment, phosphorylation and activation of TGFRI. SERT stimulation was dependent upon TGF-β1 receptor-mediated signaling, as SB-431542, a specific inhibitor of TGFRI, blocked the stimulatory effect of TGF-β1 (Fig 1C).

### PI3K mediates effects of TGF-β1 on SERT function

**Fig 2:**
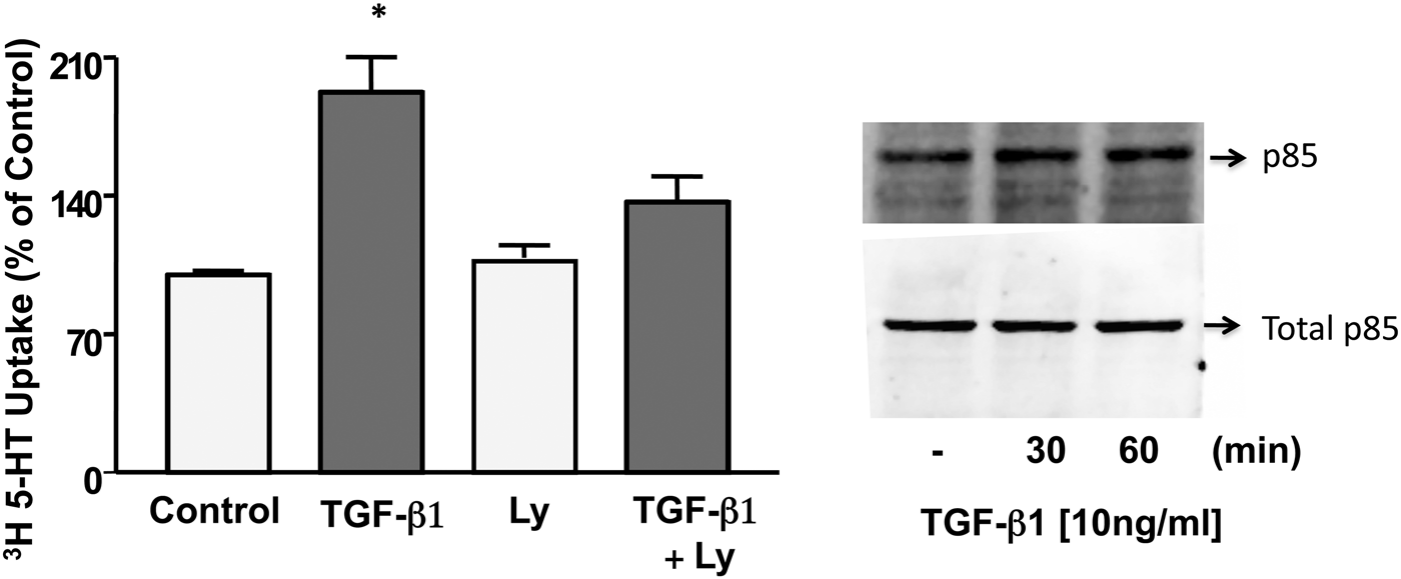
A. Ly294002 blocks the effects of TGF-β1 on SERT function. Caco-2 monolayers were pre-treated with Ly294002 (50 μM) for 30 min and then with TGF-β1 (10 ng/ml) for another 1h in the presence or absence of inhibitor. N = 3. * P<0.01 vs control. [Control Values in pmol/mg protein/5 min: 0.51 ± 0.01]. B. Phosphorylation of P85 subunit of PI3K in response to TGF-β1. Overnight serum deprived Caco-2 cells were treated with TGF-β1 for 0–60 min. Immunoblotting was performed utilizing phospho-specific P85 antibody. The blots were stripped and re-probed with the total anti-P85 antibody to normalize for equal loading of protein in each lane.

Activation of TGF-βR1 is known to activate the canonical Smad pathways, which regulate transcription of target genes. Besides the Smad pathways, non-Smad pathways including PI3K, Akt, ERK 1/2 and p38 have been shown to be principal effectors of TGF-β1. Analysis of non-Smad signaling pathways mediating the effects of TGF-β1 on SERT revealed that PI3K was involved as inhibition of PI3K pathway by specific inhibitor; LY294002 (50 μM) blocked the TGF-β1-mediated stimulation of SERT activity (Fig 2A).

This was consistent with activation of PI3K as evidenced by increase in phosphorylation of the regulatory p85 subunit in response to TGF-β1 (Fig 2B).

Densitometric analysis revealed ~1.5 fold increase in phosphorylation of p85 subunit at both 30 min and 60 min of TGF-β1 treatment [Arbitrary Units p-p85/Total p85: TGF-β1 (60 min): 150* ± 11, p<0.05 compared to control taken as 100%]. In contrast, no change in phosphorylation levels of AKT was observed (S1 Fig). These data indicate that TGF-β1 mediated stimulation of SERT activity occurs via PI3K but independent of the downstream AKT pathway.

### TGF-β1 recruits SERT-GFP to plasma membrane via PI3K

**Fig 3:**
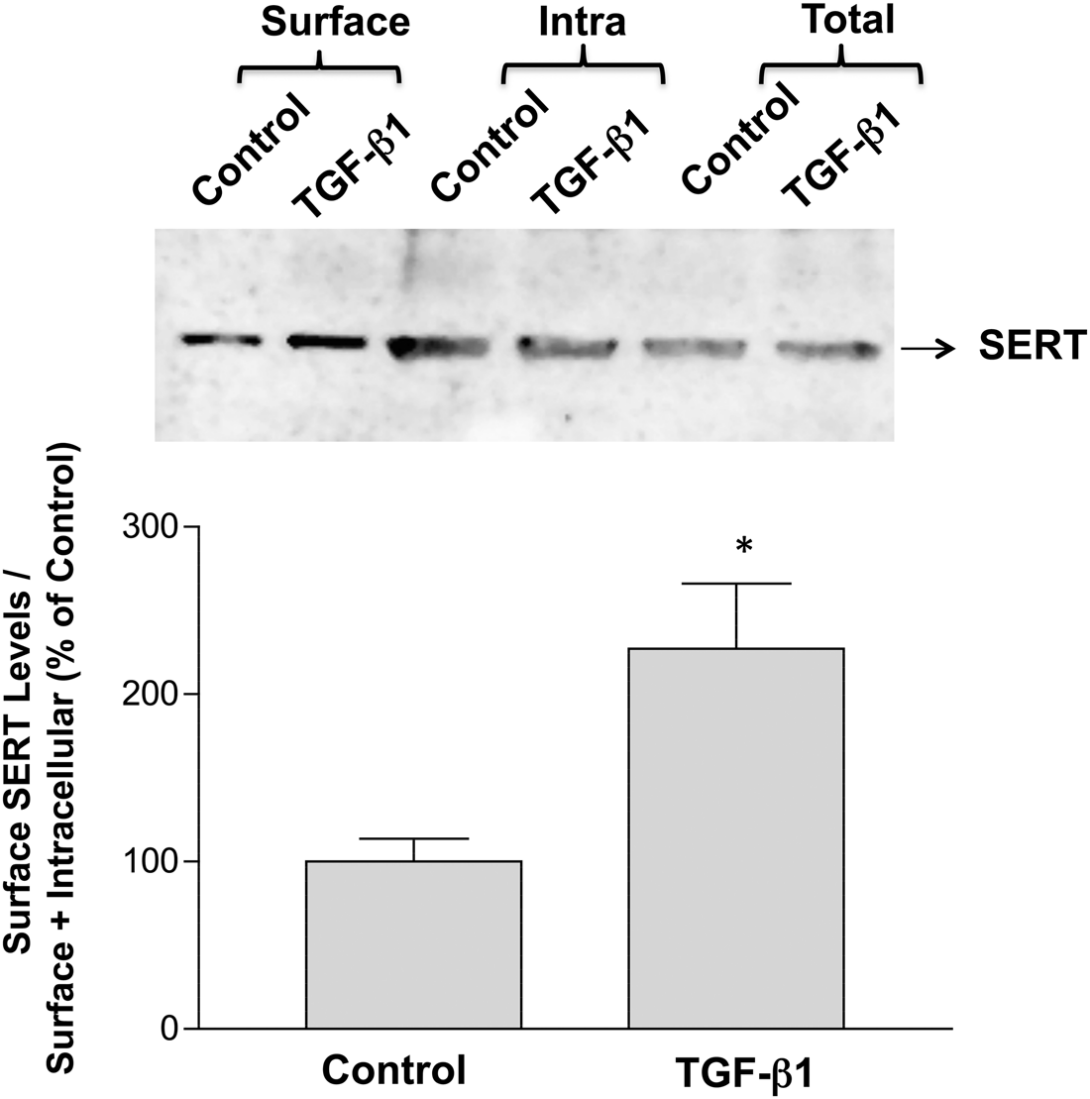
A. TGF-β1 increases surface expression of SERT in Caco-2 cells. Biotinylated proteins were run on SDS-polyacrylamide gel. The blot was immunostained with anti-SERT antibody. Representative blot of 3 separate experiments is shown. B. Densitometric analysis of SERT surface expression. Results are expressed as surface SERT/ total SERT (Surface + Intracellular). Values represent mean ± SEM of 3 different experiments. *p<0.05 or less compared to control.

To examine mechanisms underlying TGF-β1 mediated increase in SERT function, cell surface biotinylation studies were performed to determine the changes in surface SERT, identified as the biotin-accessible fraction of total cellular SERT levels. Biotinylated proteins from control and treated cells were separated from the cell lysate by avidin and proteins were probed with affinity purified human anti-SERT antibodies. SERT surface expression in TGF-β1 treated cells was considerably increased paralleling the loss in intracellular fraction, while total cellular SERT (sum of apical and intracellular) remained unchanged (Fig 3A). Densitometric analysis suggested that TGF-β1 increased surface SERT expression by >2 fold compared to control (Fig 3B).

**Fig 4:**
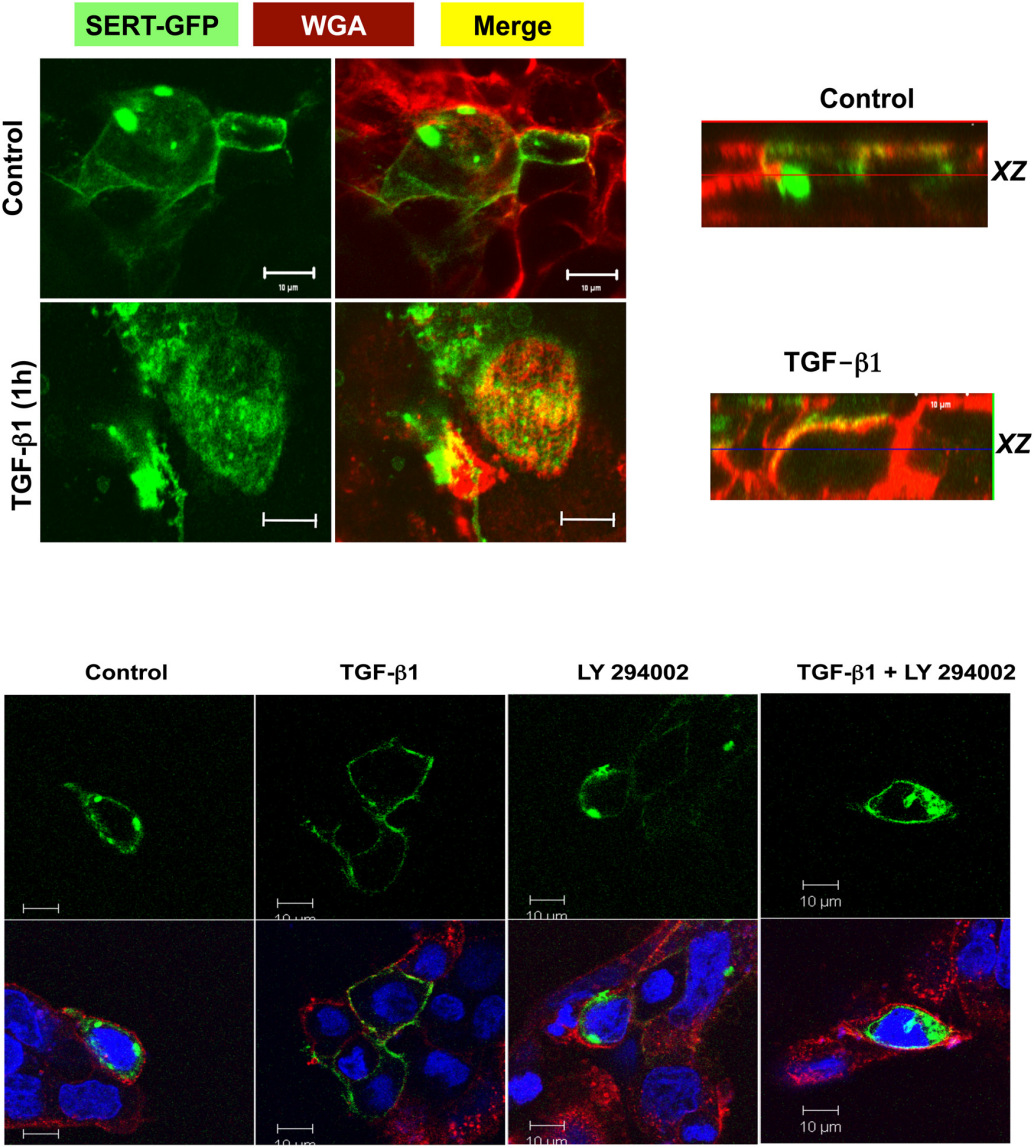
A. Short-term treatment of Caco-2 cells with TGF-β1 increases SERT plasma membrane expression. Cells were transfected with SERT-GFP vector and 48h post-transfection were treated with TGF-β1 (10 ng/ml). Microscopy was performed and a series of sections (0.5 μm) were taken at z direction and orthogonal sections were made in a z stack. Arrows indicate SERT vesicles. SERT GFP (green), WGA (red), Blue (Nuclei) Merge (yellow). B. TGF-β1 mediated recruitment of SERT to plasma membrane is PI3K dependent. SERT-GFP transfected cells were pre-treated with specific PI3K inhibitor Ly294002 (50 μM) for 30 min and then with TGF-β1 (10ng/ml) for another 1h in the presence or absence of inhibitor. Cells were fixed, counterstained with WGA and DAPI and microscopy was performed.

Cell surface biotinylation was complemented by confocal microscopy studies in which SERT-GFP construct was transiently transfected in Caco-2 cells and SERT cellular localization was visualized by confocal microscopy in response to TGF-β1. Cells were also labeled with lectin wheat germ agglutinin (WGA) to demarcate the boundary of cells. As shown in Fig 4A, WGA counterstained the plasma membrane of the cells (red). In untreated cells, SERT-GFP was present as vesicles in the intracellular compartments with some expression on the membrane. Similar to surface biotinylation results, TGF-β1 treatment decreased the expression of SERT-GFP in the intracellular compartments, concomitant with increased SERT co-localization with WGA on the apical side as judged by both the vertical *xy* image (yellow) and orthogonal images (*xz*) (Fig 4A). Specific inhibition of PI3K by LY294002 attenuated the increase in the surface expression of SERT-GFP in response to TGF-β1 (Fig 4B). These data indicate that TGF-β1 recruits SERT to the plasma membrane by activation of PI3K to stimulate SERT function possibly via trafficking dependent mechanism

### Role of exocytosis in increased SERT function by TGF-β1

**Fig 5:**
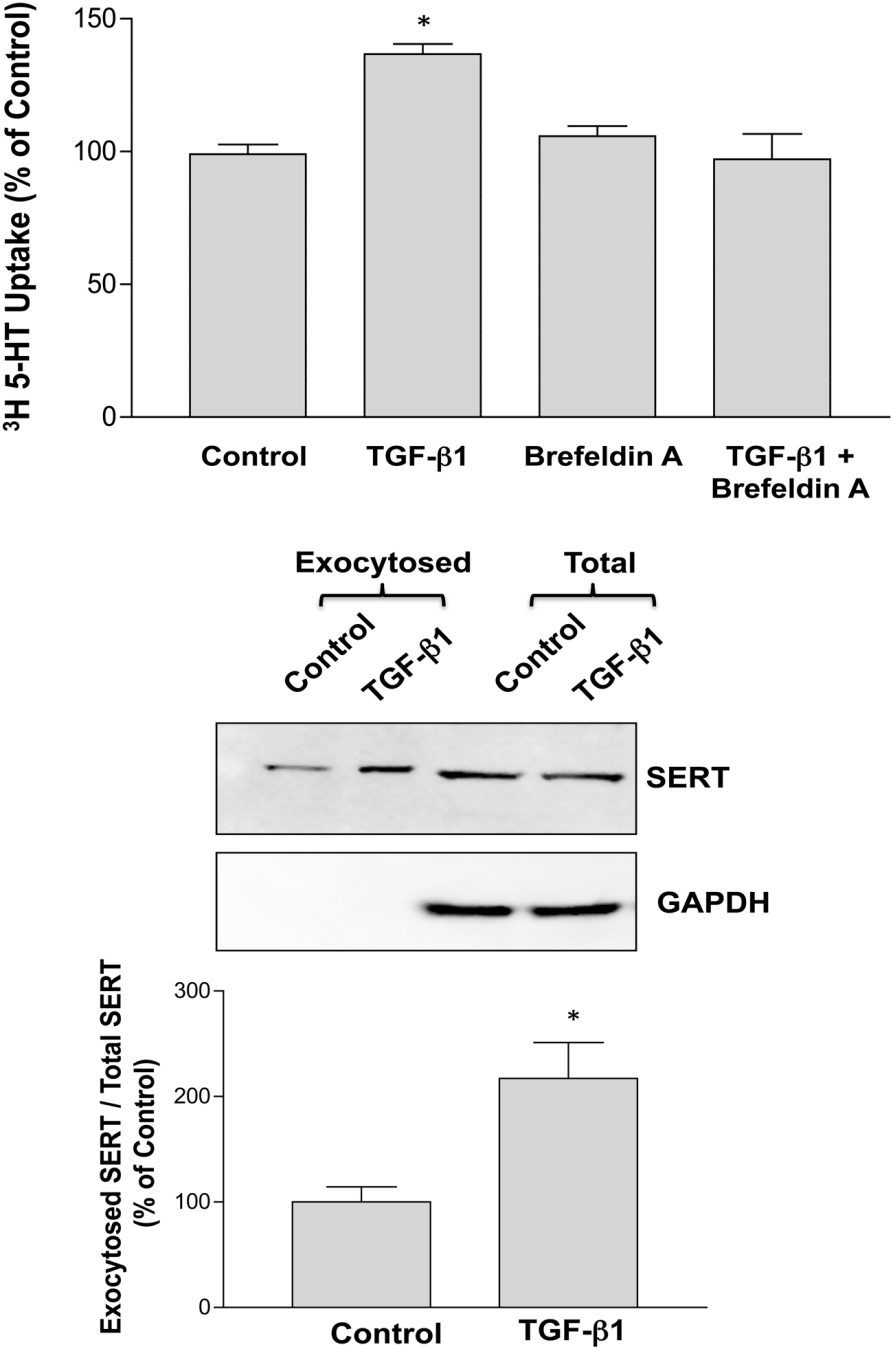
A. Brefeldin A attenuates TGF-β1 induced increase in SERT function. Caco-2 monolayers were pre-treated with specific exocytosis inhibitor Brefeldin A for 30 min and then with TGF-β1 (10ng/ml) for another 1h in the presence or absence of inhibitor and SERT function was assessed from apical side. N = 3. * P<0.01 vs control. B. Exocytosis of SERT in response to TGF-β1. Surface NHS reactive sites on Caco-2 cells grown on Transwells were masked with NH-SS-Acetate at 4°C for 1 hour. Cells were then treated with TGF-β1 for 60 minutes at 37°C and the amount of protein exocytosed to the surface was measured by cell-surface biotinylation using sulfo-NH-SS-S-biotin at 4°C for 1 hour. Results are expressed as exocytosed SERT/total SERT and values are mean ± SEM of 3 different blots. ***p<0.005 as compared to control.

The increase in SERT function and surface levels by TGF-β1 may occur via membrane trafficking events such as alteration in the endocytosis or exocytosis of SERT to the plasma membrane. To check this possibility, we first used Brefeldin A (BFA), a fungal metabolite demonstrated to reversibly interfere with anterograde transport from the endoplasmic reticulum to the Golgi apparatus. As shown in Fig 5A, treatment with BFA had no affect on basal SERT function, however, the TGF-β1 stimulated increase in SERT activity was abrogated in the presence of BFA. These data indicate that TGF-β1 stimulates SERT function by increasing exocytosis of SERT protein.

To directly examine the possibility of SERT exocytic insertion, we incubated cells with sulfo-NHS-acetate to saturate NHS-reactive sites on the cell surface. Cells were then warmed to 37°C for 1h in the presence or absence of TGF-β1 to permit SERT recycling, surface labeled with sulfo-NHS-SS-biotin, lysed and biotinylated fraction representing newly inserted surface SERT proteins was measured by western blotting. No expression of GAPDH was observed in exocytosed fraction indicating that only plasma membrane proteins were biotinylated. SERT expression in the exocytosis pool was considerably enhanced by TGF-β1 (Fig 5B). Densitometric analysis revealed ~2 fold increase in SERT levels in the exocytosed pool relative to total SERT levels (Fig 5B).

### Interaction of Syntaxin 3 (STX3) is enhanced by TGF-β1

**Fig 6:**
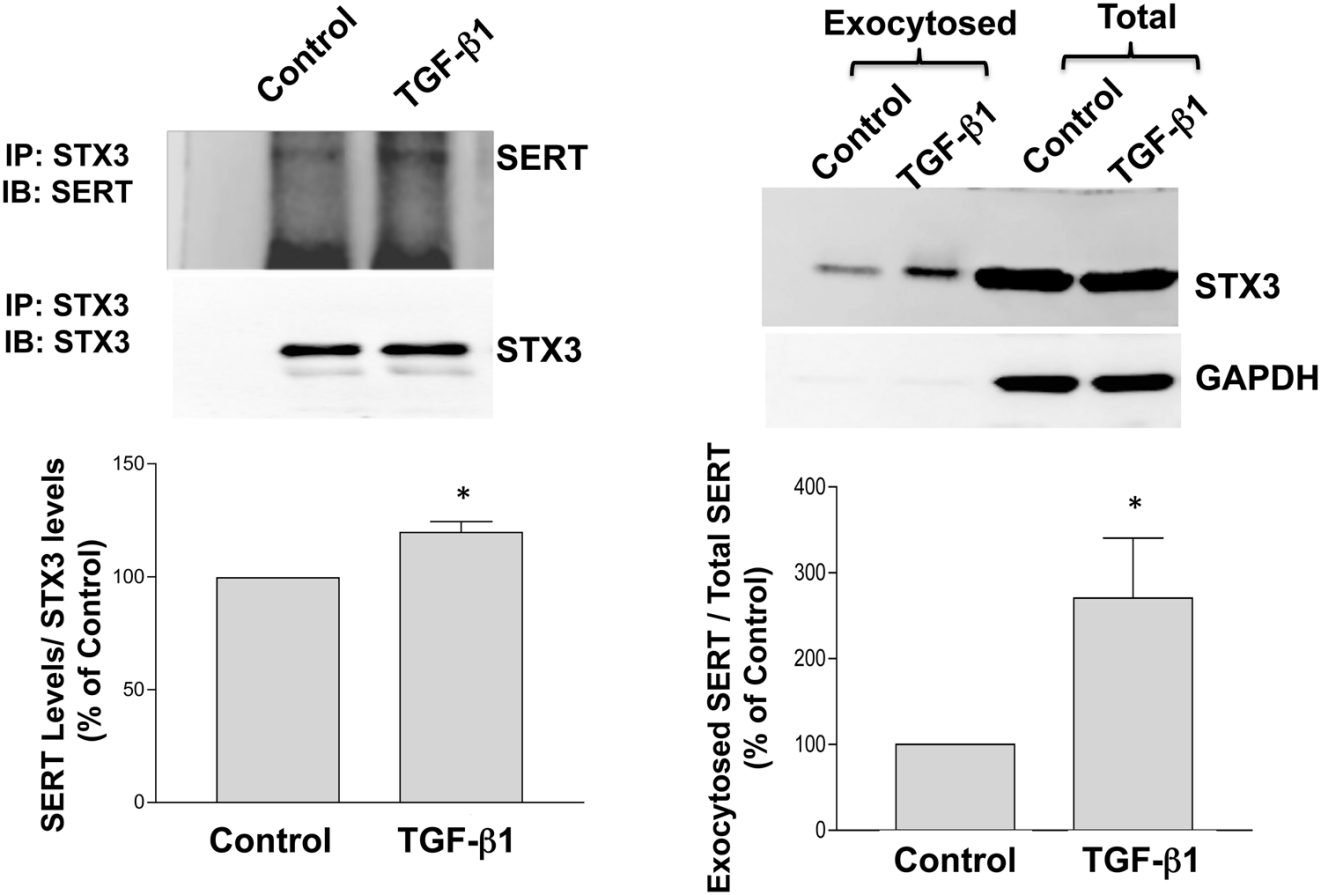
A. Association of SERT with Syntaxin 3 is enhanced by TGF-β1. Immunoprecipitates were analyzed by 10% SDS-polyacrylamide gel electrophoresis, followed by transfer of proteins to nitrocellulose and probed with anti-SERT antibody. The blots were stripped and reprobed with the anti-STX3 antibody to normalize for equal loading of protein or equal input in each lane. A representative blot of 3 different experiments is shown. Densitometry analysis was performed for quantification of data expressed as arbitrary units (A.U.). ***p<0.005 as compared to control.*P<0.05 compared to control. B. TGF-β1 increases exocytosis of STX3: Results are expressed as exocytosed STX3 / total STX3 and values are mean ± SEM of 3 different blots. ***p<0.005 as compared to control.*P<0.05 compared to control.

We next examined the mediators of TGF-β1 mediated SERT exocytosis. The role of the SNARE (Soluble N-ethyl-maleimide sensitive factor Attachment protein Receptor) family of integral membrane proteins was first examined [23]. Previous studies have shown that t-SNARE, STX1a interacts with SERT in the neuronal cells [24, 25]. However, STX1A has been shown to exhibit negligible expression in intestinal epithelial cells such as Caco-2 [23]. Consistent with these data, Caco-2 cells under the conditions of our study did not show expression of STX1A (data not shown). Syntaxin 3 (STX3), the exocytic protein involved in vesicle fusion has been shown to be abundantly expressed apically in IECs [26]. To examine whether STX3 interacts with SERT, co-IP studies were performed. SERT was immunoprecipitated with specific antibody and probed for STX3. Data demonstrated that STX3 interacted with SERT under basal conditions and this association was enhanced that TGF-β1 treatment in Caco-2 cells (Fig 6A).

Similar to SERT, exocytosis of STX3 was also increased by TGF-β1 indicating that both proteins exist in the same pools of exocytotic machinery (Fig 6B).

### TGF-β1 increases SERT cellular trafficking in 3D cell culture model

**Fig 7:**
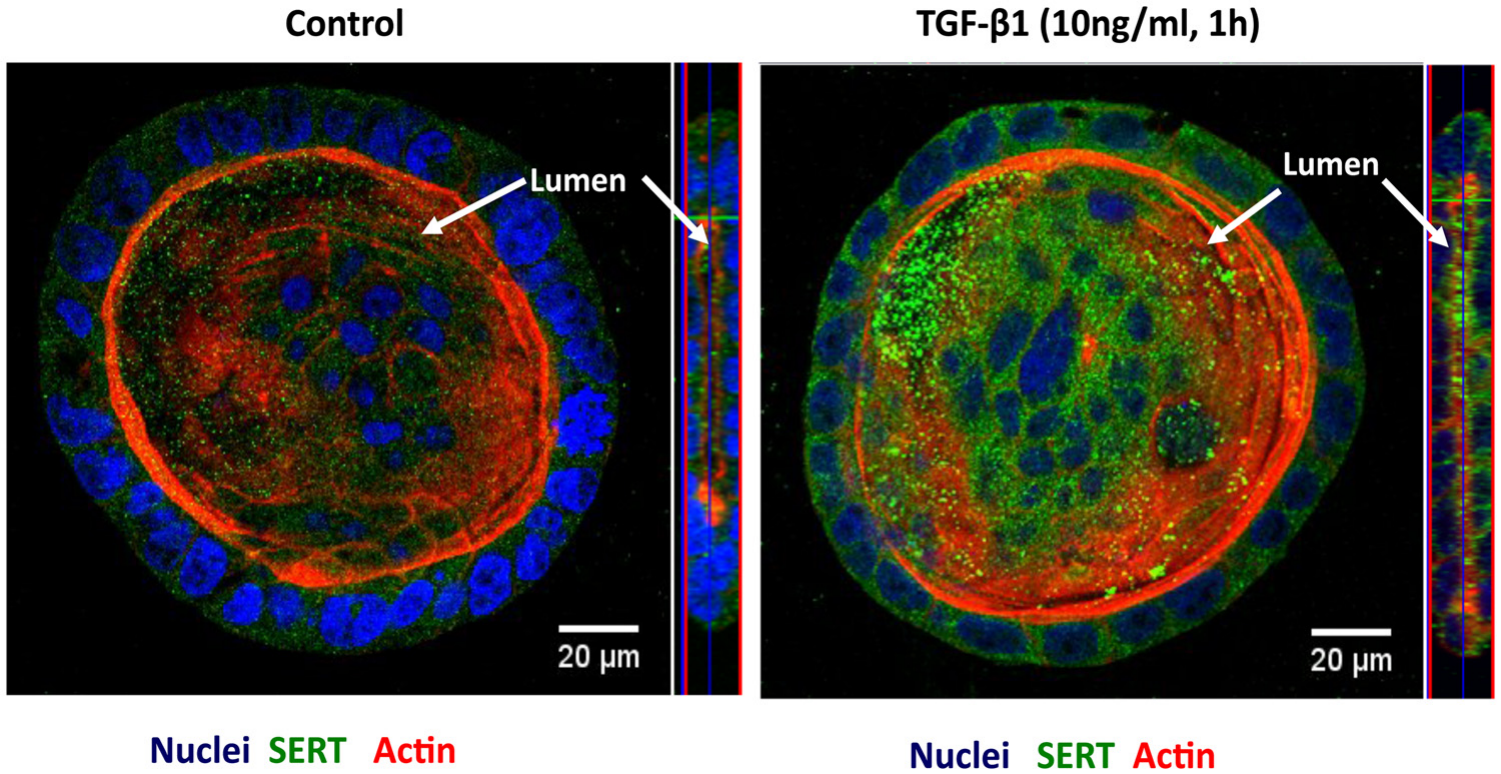
TGF-β1 increases SERT levels on the luminal surface in 3D-culture of Caco-2 cells. Caco-2 cells in 3D culture system untreated or treated with TGF-β1 (10 ng/ml, 1h) were stained for SERT (green), Phalloidin (red) and DAPI (blue) and visualized by confocal microscopy. XY planar images and orthogonal XZ images Orthognal *xz* images were obtained with a Zeiss LSM 510 confocal microscope.

Although 2-dimensional cell culture systems have been extensively used to study ion transporters, one of the limitations in this model system is that the cells are forced to adapt to an artificial, flat and rigid surface. To further substantiate our results in a more physiological setting, we utilized the three-dimensional (3D) Caco-2 culture system. At 12 days, Caco-2 cells formed cysts containing lumen in a matrigel-based 3D culture as shown in Fig 7.

To determine the short-term effects of TGF-β1 on SERT expression, Caco-2 cells in 3D were treated with 10ng/ml TGF-β1 for 1h from basolateral side. After fixing the cells in matrigel, cysts were stained with SERT antibody (green) and with phalloidin to stain actin (red). TGF-β1 treatment increased SERT surface expression along the luminal side (Fig 7) as evidenced by planar and orthogonal views as compared to control cells. It was intriguing that total SERT expression also appeared more in TGF-β1 treated samples. However, these images provided only qualitative data. Quantification of the total SERT expression in control and TGF-β1 treated cysts demonstrated no alteration in SERT mRNA expression in response to 1h TGF-β1 treatment (S2 Fig). These results further indicate that endogenous SERT is regulated by TGF-β1 via trafficking mechanisms that facilitate SERT translocation to apical membranes.

### *Ex vivo* TGF-β1 treatment increases SERT function in mouse intestine

**Fig 8:**
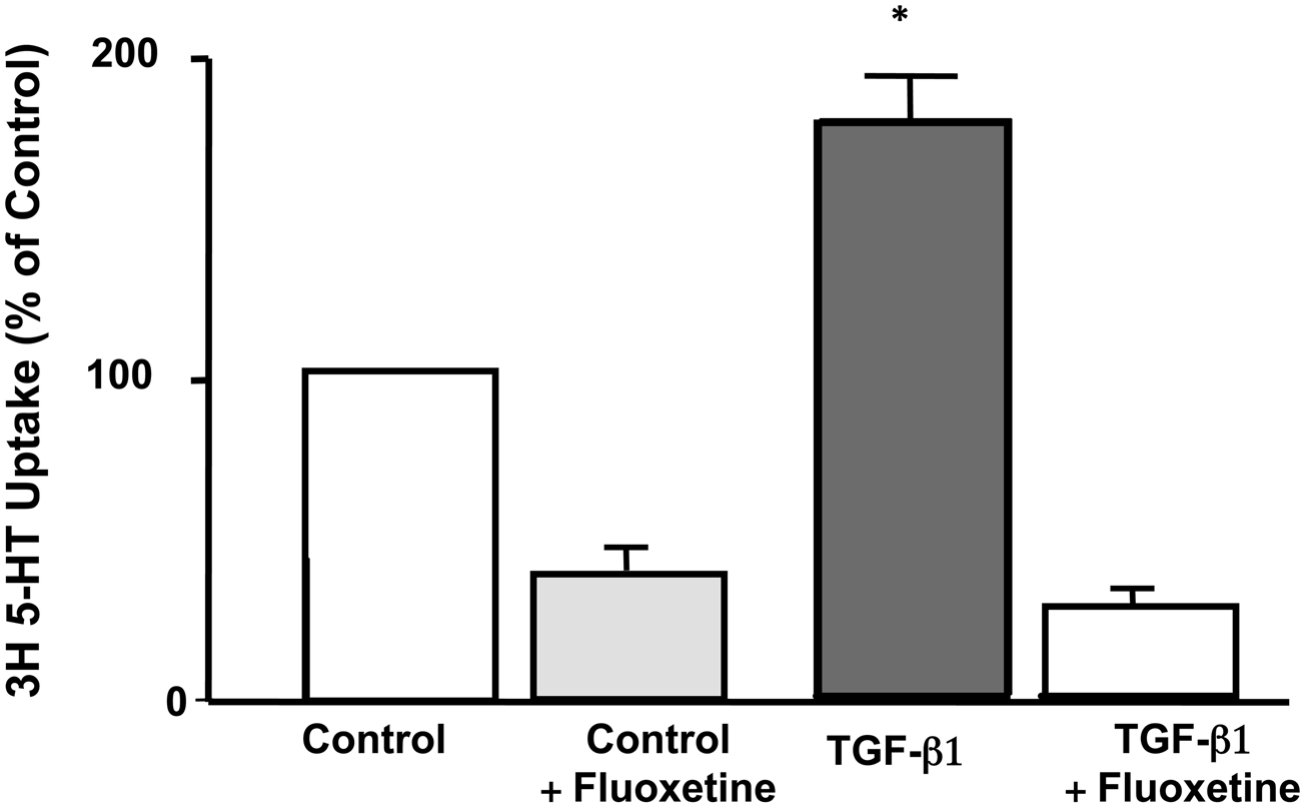
SERT function in response to TGF-β1 treatment of native mouse ileum. The distal small intestine was stripped off of the muscle layer, mounted in the Ussing chamber to expose tissue on both sides to Kreb’s solution supplemented with L-ascorbic acid (100 μM) and the monoamine oxidase inhibitor pargyline (100 μM). Tissues were treated with TGF-β1 for 1h from basolateral side and then incubated with ^3^[H]-5-HT for 30 min. Fluoxetine (10 μM) was added 30 min prior to TGF-β1 treatment from the apical side. Values represent mean ± SEM of 3 different experiments. *P<0.05 compared with untreated control.

To validate the direct effects of TGF-β1 on SERT function in the native intestine, the *ex vivo* effects of short-term TGF-β1 treatment on SERT function were next examined in mouse ileum. Ileal mucosa stripped of the muscle layer was mounted in Ussing chamber and measurement of fluoxetine-sensitive ^3^[H]-5-HT uptake was done in response to control or TGF-β1 treatment. TGF-β1 treatment (10 ng/ml, 1h) significantly increased SERT function as evidenced by ^3^[H]-5-HT uptake. Treatment of the ileal tissue with the Selective serotonin reuptake inhibitor, fluoxetine inhibited both basal and TGF-β1 stimulated 5-HT uptake indicating that uptake is specific to SERT (Fig 8).

## Discussion

The prevailing treatments of diarrhea and inflammatory bowel diseases are limited warranting improved targeted therapies. Increasing evidence implicates a decrease in intestinal 5-HT transporter (SERT) and the consequent high 5-HT levels in the pathophysiology of intestinal disorders [15, 27–32]. Thus, SERT is an attractive target for the treatment of gut disorders. Thus, appropriate regulation of SERT is critical for the maintenance of normal 5-HT homeostasis in health and disease. Current study for the first time demonstrated that TGF-β1 rapidly stimulates SERT function by increasing its exocytosis via TGF-β1 receptor mediated activation of non-smad pathways in intestinal epithelial cells.

Short-term stimulation of SERT may involve multiple mechanisms including alterations in SERT phosphorylation and/or its membrane levels. Previous studies utilizing transfected HEK-293 cells revealed SERT to be a phosphoprotein where phosphorylation was shown to occur with PKC activation or protein phosphatase 2 A inhibition [33]. However, detailed characterization of SERT recycling and signaling pathways in Intestinal epithelial cells is lacking. Utilizing the well-established in vitro model epithelium, Caco-2 monolayer’s, current findings revealed an important role of PI3K in TGF-β1 mediated stimulation of SERT activity. PI3K regulates a number of signal transduction pathways including the protein serine/threonine kinases Akt and PDK1, protein tyrosine kinase exchange factors for GTP-binding proteins (Grp1 and Rac exchange factors) adaptor proteins (GAB-1)[34]. In addition, numerous studies have provided significant insights into the roles of PI3-kinases, their catalytic products and their downstream effectors in membrane trafficking [35]. PI3K pathway can influence cellular functions through AKT-dependent or AKT independent mechanisms [36, 37]. Delineation of cellular mechanisms underlying PI3K mediated stimulation of SERT function reveled that the effects of TGF-β1 were AKT independent as no phosphorylation of AKT was observed in response to TGF-β1. Our data further demonstrated that TGF-β1 increases the recruitment of SERT on the apical plasma membrane and provided mechanistic link that PI3K is necessary for this apical recruitment. The increase in surface expression of SERT occurred because of increased exocytic retrieval of SERT by TGF-β1. In this regard, the role of PI3K in regulated exocytosis has been previously established [38]. For example, PI3K has been implicated in insulin-induced membrane translocation of GLUT4 glucose transporters [39] neurosecretory granule exocytosis [40] and angiotensin II dependent regulation of exocytosis of the Na^+^/H^+^ exchanger 3 [41], EGF/NGF/IGF-1 regulation of insertion of TRP (transient receptor potential) family ion channels. Our studies, however, do not rule out the role of other non-Smad pathways such as p38 or Erk1/2 in TGF-β1 mediated effects on SERT.

With respect to mediators of exocytosis, the association of the plasma membrane SNARE protein STX1A with a number of neurotransmitter transporters including GAT-1 (GABA transporter 1), NET (norepinephrine transporter) and neuronal SERT has been previously reported. For example, studies have shown that incubation of thalamocortical cultures with botulinum toxin C1, which specifically cleaves STX1A, decreased neuronal SERT function [24, 25]. STX1A expression, however, is detected to a much lesser extent than brain in intestinal epithelial cells such as in colonic HT-29-CL109 cells [42] and is not detected in Caco-2 cells [23]. In contrast to STX1A, STX3 has been shown to be abundantly expressed in IECs [23] and plays important role in membrane polarity, apical targeting, recycling and vesicle fusion in the intestine [26]. Syntaxin 3 protein has been precisely localized to the apical cell surface in epithelial cells such as MDCK and Caco2 cells [26]. Previous studies have implicated STX3 in regulated exocytosis of CFTR [43] and in cAMP induced acid secretion [44]. Since SERT is a pharmacological target for both brain and gut disorders, it was plausible to speculate that distinct cellular mechanisms govern SERT function and membrane trafficking in neurons versus gut. Interestingly, our data demonstrated that exocytosis of both SERT and STX3 was increased by TGF-β1 indicating that both proteins exist in the same pools of exocytotic machinery. Consistently, immunoprecipitation studies showed that STX3 interacts with intestinal SERT under basal conditions and this association was enhanced by TGF-β1 treatment in Caco-2 cells.

In addition to elucidation of important SERT regulatory mechanisms in the IECs, our findings for the first time provide feasibility of utilizing 3D-culture system of Caco-2 cells for studying endogenous SERT regulation. Acute treatment with TGF-β1 did not alter the total SERT expression in 3D-culture (S2A Fig); however, the increase in SERT staining was more apparent on the luminal side. When compared to the traditional 2D system, SERT protein expression in untreated cells was significantly higher in the cells grown as cysts with clearly visible lumen at 12 d (S2B Fig and S2C Fig). Recent studies from our group have demonstrated that the 3D-system is more suitable model to examine responsiveness of ion transporters to pathophysiological stimuli because of higher expression of critical signaling components [45]. SERT has been previously shown to be inhibited by the pro-inflammatory mediators, LPS and TNF via distinct mechanisms. For example, lipopolysacchardie (LPS) decreased SERT levels at the plasma membrane [46], whereas, TNF reduced SERT mRNA expression [47], albeit the detailed mechanisms underlying these effects are not known. Future studies will examine if TGF-β1 induced Smad and non-Smad pathways counteract down-regulation of SERT in response to TNF or LPS in 3D culture, which may underlie its beneficial effects in alleviating diarrhea or intestinal inflammation.

Interestingly, large quantities of TGF-β1 are normally produced in the GI tract, although, in a latent precursor form, requiring cleavage and dissociation for conversion to a mature bioactive dimer [48]. TGF-β1 is also abundant in human milk and active TGF-β1 moieties are released via passage through acidic pH of stomach [49]. To further validate the short-term regulation of SERT by TGF-β1 in the native intestine, we utilized ex vivo mouse model system. Similar to in vitro results, TGF-β1 treatment of the muscle stripped ileal mucosa mounted in Ussing chambers increased fluoxetine-sensitive Na^+^-Cl^-^ dependent ^3^H[5-HT] uptake. These studies revealed that although TGF-β1 targets could be multiple, it directly modulates SERT in the native intestine.

In summary, the findings presented here increase our understanding of the cellular mechanisms increasing SERT function that may identify potential therapeutic approaches to manage GI disorders where SERT is dysregulated (such as infectious diarrhea and IBD). The role of canonical Smad pathways and their cross-talk with early non-Smad pathways delineated here in modulation of SERT by TGF-β1 will be the subject of future investigations.

## Supporting Information

S1 Fig No alteration in phosphorylation of AKT in response to TGF-β1 (1h) treatment to Caco-2 cells. (TIF)

S2 Fig A. Relative SERT mRNA levels in TGF-β1 treated Caco-2 cells. B. Fig. SERT protein expression in 2D Caco-2 cell monolayers vs 3D Caco-2 cysts grown on matrigel. C. Fig. Densitometric analysis showing relative SERT protein levels normalized to GAPDH. *p <0.05 vs Control.
(TIF)
