# Supplementary figures and images for: Mechanisms of Intestinal Serotonin Transporter (SERT) Upregulation by TGF-β1 Induced Non-Smad Pathways
Source: PLoS One. 2015 May 8;10(5):e0120447. doi: 10.1371/journal.pone.0120447 (PMC4425666; doi:10.1371/journal.pone.0120447)

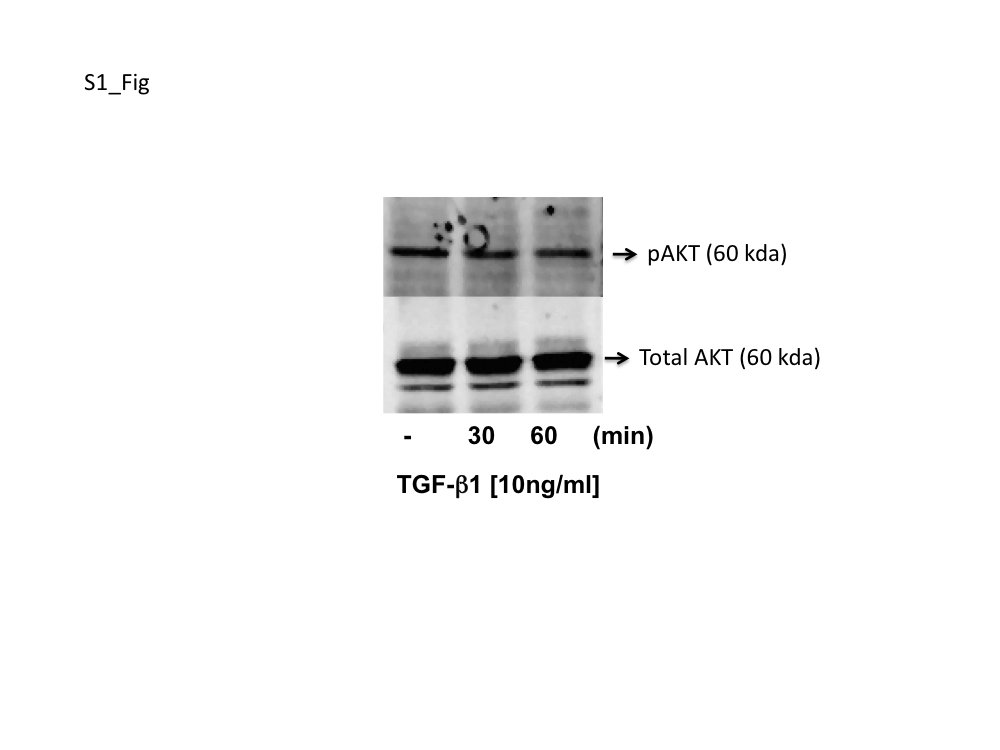

Supplement: S1 Fig — (TIF) [file pone.0120447.s001.tif]

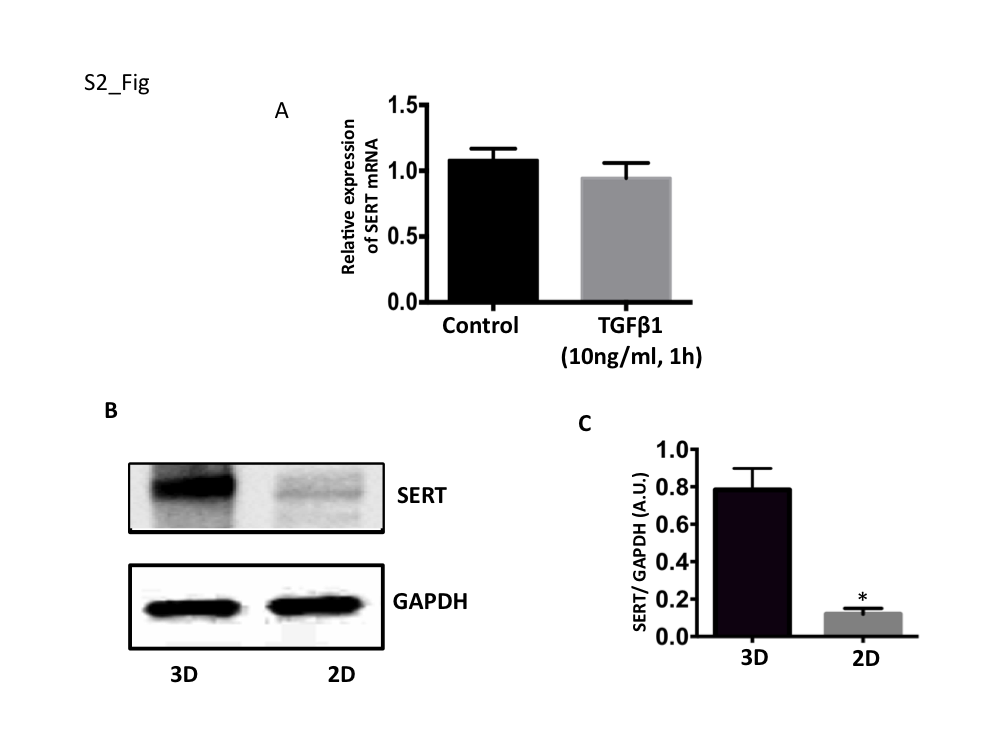

Supplement: S2 Fig — B. Fig. SERT protein expression in 2D Caco-2 cell monolayers vs 3D Caco-2 cysts grown on matrigel. C. Fig. Densitometric analysis showing relative SERT protein levels normalized to GAPDH. *p <0.05 vs Control. (TIF) [file pone.0120447.s002.tif]
